# Genito Urinary and Syphilitic Diseases

**Published:** 1896-12

**Authors:** Byron H. Daggett

**Affiliations:** Buffalo, N. Y.


### Progress in Medical Science.

### GENITO-URINARY AND SYPHILITIC DISEASES.

CONDUCTED BY BYRON H. DAGGETT, M. D., Buffalo, N. Y.

### DIAGNOSIS OF CALCULUS BY THE NEW PHOTOGRAPHY.

CHOPPINS (*Medical Bulletin* for September, 1896,) shows that it will soon be possible to diagnose calculi with absolute exactness. While their pictures do not show the organ in which the calculus is located, they make it possible to distinguish the substance of which the calculus is composed, whether it is homogeneous or formed of different layers, whether it is large or small. The differences in the depth of the shadow in the photograph are so marked as to indicate the characteristics and kind of calculi. The tiny kernel of uric acid is distinctly visible, while the outer layers of the larger calculus show distinctly defined rings. One photograph showed a kernel of urate of soda in an outer layer of triple phosphate clearly defined.

Photograph showed : (1) The silhouette cast by a calculus of pure uric acid. (2) Calculus of the same size composed of phosphate of ammonia and magnesia. (3) A much larger calculus of several distinct layers of uric acid in the center, with an outer layer, entirely different in color, and composed with triple phosphate. Another photograph showed several uric acid calculi lodged in the parenchyma of the kidney. The rays passed through this thick, tough tissue, photographing only the calculi.

There is a method described in the *Semaine Medicale*, whereby, instead of taking photographs, we may look in and see the skeleton with our own eyes. Buka, Röntgen, Salvioli, Lewy, du Bois-Raymond and others have distinguished the skeleton and organs throughout the body and diagnosticated, with great accuracy, several cases of arterio-sclerosis and the like by this method.

Becker, of Berlin, suggests that lime water be injected into the hollow organs of the body, or that air be introduced so that the organ may be seen or photographed. The lime makes fluorescence and the air permits the passage of the Röntgen rays. This new photography will enable us to diagnosticate and locate calculi and outline the viscus containing it.

---

### FORMALIN IN DISEASES OF THE GENITO-URINARY TRACT.

LAMORQUE (*Ann. d. Mal. d. Org. Ur.*, 1895, p. 910,) states that he has tried formalin in acute and chronic gonorrhea and in cystitis in various forms.

He used it by irrigation, 1 part to 500 of water, and by instillation, 1-100, employing a 40 per cent. solution of the French drug in alcohol. The results were no better than those produced by other well-known drugs, except in tubercular cystitis it promises to be of real value. It was used in five cases of tubercular cystitis, in which bichloride and iodoform instillations had been used without much benefit.

It induced marked diminution of all the symptoms. It should be used at least every other day. Pain was obviated by a previous injection of a solution of antipyrine.

---

### QUICK FILTRATION OF URINE.

A SMALL quantity of urine (*Boston Medical and Surgical Journal*) is placed in a test tube and the mouth is plugged with cotton. A second tube is placed over the first, mouth to mouth, and then the double tube is reversed in position. The upper tube containing the urine is gently heated, so that the expansion of the air forces the urine through the cotton and the filtered urine collects in the lower tube.

---

### SIMPLE TEST FOR ALBUMIN.

Dr. A. C. Ewing (*Medical Record*) says: Draw up into a small glass pipette or tube about an inch of the urine, let the finger remain tightly over the top and insert the pipette into nitric acid and draw up under the urine about the same quantity of acid, when, if even a trace of true albumin be present, there will appear a beautiful line of demarcation between the acid and urine. This test is as accurate as it is simple, and, besides, is decidedly economical and far less troublesome than any of the other tests.

---

### RESULTS OF CASTRATION UPON PROSTATIC HYPERTROPHY, AS SHOWN IN SIX CASES.

Dr. L. S. Pilcher (*Annals of Surgery*, XXIII., No. 6, p. 687,) reports four prostatectomies resulting in death of all of the subjects within ten days.

Dr. Pilcher also reports six orchidectomies for the relief of urinary disturbance and obstruction, caused by senile hypertrophy of the prostate. In all of these cases there was a permanent improvement in the urinary obstructive symptoms and in most of them marked improvement in the general health. In one case nocturnal delirium and impairment of the mental faculties developed three months after the operation.

---

### CASTRATION FOR ENLARGED PROSTATE.

Cabot, of Boston, reviewing this subject, formulates the following conclusions: 1. Castration is quite as fatal a procedure as prostatectomy. 2. Prostatectomy has an advantage in that it opens a way to examine the bladder, and, perhaps, save a second operation. Calculi have been found in the bladder, following castration for the relief of prostatic hypertrophy. 3. Prostatectomy confines the patient a longer time and is liable to be followed by fistula. 4. It is too early to know whether permanent loss of vigor follows castration in old men. 5. The functional results and relapses are about equal in these procedures. 6. Castration diminishes congestion, thus reducing the size of the prostate, followed by degeneration, absorption and atrophy of the glandular elements. 7. Castration is especially indicated in cases of large prostates, when urinary obstruction is due to pressure of the lateral lobes. 8. Castration is of little use in myomatous and fibrous prostates. 9. Prostatectomy is indicated for the relief of valvular obstructions. A skilful operator will correct any form of prostatic obstruction by prostatectomy. 10. Prostatectomy is applicable to more cases than castration. It permits diagnosis, operation and drainage.

---

### ARGONIN FOR GONORRHEA.

SWINBURNE, New York, (*Jour. Cutaneous and Genito-urinary Diseases*, August, 1896,) relates his experience with argonin in the treatment of acute gonorrhea. This drug is a compound of silver and casein, forming a grey-white powder, 15 to 1 of silver. It is stated that chlorate of sodium does not precipitate the silver, and albuminous substances do not decompose the compound.

Jodasson alleges that it possesses powerful germicidal properties, that it is not irritating even in concentrated solutions, and that it possesses no astringent properties. It does not stain or discolor clothing or skin. Heated over a water-bath in water it forms an opalescent, viscid, albuminous fluid, neutral in reaction. The maximum strength is 10 per cent.

Dr. Swinburne states that a 10 per cent. solution of argonin, by injection, caused a rapid diminution of the discharge and of the number of gonococci. In some cases the germs were rather persistent and sometimes increased in numbers. In several instances they reappeared after omission of the treatment, but were quickly eliminated by its renewal. This treatment caused no inflammatory reaction ; on the contrary, it diminished the inflammatory action caused by the disease. In the earliest stages of the disease the ardor urinæ is markedly diminished or completely relieved by this treatment.

When posterior urethritis was present, the doctor injected two drachms of a 10 per cent. solution through a soft catheter passed through the constrictor muscle into this region. In all cases he first irrigated with a solution of permanganate of potash. Injections of argonin (10 per cent.) caused no pain, only "a little burning for a while."

The reviewer insufflates the posterior urethra by means of a Codman & Shurtliff powder blower and a urethral tube, fenestrated at its distal end, applying in this way full strength argonin powder to the dilated posterior urethra. Results have been very satisfactory. This powder is not to be repeated too often or continued too long.

Dr. Swinburne further says that he has used argonin in about a dozen cases of chronic urethritis, which were rebellious to other modes of treatment, and has been impressed with the results. So say we, also.

Jodasson says that the addition of 0.3 per cent. of liquor ammoniæ caustici increases the penetrating power of the solution.

Dr. A. Lewin, in the *Berliner Klinische Wochenschrift* (*Medical Bulletin*), says that he has employed argonin in solution, three parts to 200, and injected ten centimeters five times daily, which were held in the urethra five minutes.

Of the twelve cases reported, in nine the gonococci disappeared in from two to six days ; one case, after four weeks' treatment with injectio composito, still showed abundant gonococci ; was freed from these germs by two days' treatment with argonin. In another case, gonococci were found after ten days' treatment with argonin ; in still another, gonococci reappeared as soon as the argonin treatment was interrupted. Lewin says there was not a single complaint of irritation. He could increase this dose largely and not have complaints.

If the secretion persisted after discontinuing the argonin, Dr. Lewin says that the use of sulpho-carbolate of zinc will quickly check it. Dr. Lewin agrees with Jodasson that argonin possesses preëminent gonococci-destroying qualities, and does not cause irritation.

